# Finding the needle in the haystack—An interpretable sequential pattern mining method for classification problems

**DOI:** 10.3389/fdata.2025.1604887

**Published:** 2025-10-24

**Authors:** Alexander Grote, Anuja Hariharan, Christof Weinhardt

**Affiliations:** Institute for Information Systems (WIN), Karlsruhe Institute of Technology (KIT), Karlsruhe, Germany

**Keywords:** sequential pattern mining, feature selection, sequence classification, interpretable machine learning, categorical time series

## Abstract

**Introduction:**

The analysis of discrete sequential data, such as event logs and customer clickstreams, is often challenged by the vast number of possible sequential patterns. This complexity makes it difficult to identify meaningful sequences and derive actionable insights.

**Methods:**

We propose a novel feature selection algorithm, that integrates unsupervised sequential pattern mining with supervised machine learning. Unlike existing interpretable machine learning methods, we determine important sequential patterns during the mining process, eliminating the need for post-hoc classification to assess their relevance. Compared to existing interesting measures, we introduce a local, class-specific interestingness measure that is inherently interpretable.

**Results:**

We evaluated the algorithm on three diverse datasets - churn prediction, malware sequence analysis, and a synthetic dataset - covering different sizes, application domains, and feature complexities. Our method achieved classification performance comparable to established feature selection algorithms while maintaining interpretability and reducing computational costs.

**Discussion:**

This study demonstrates a practical and efficient approach for uncovering important sequential patterns in classification tasks. By combining interpretability with competitive predictive performance, our algorithm provides practitioners with an interpretable and efficient alternative to existing methods, paving the way for new advances in sequential data analysis.

## 1 Introduction

Sequential pattern mining (SPM) is a critical area of data mining, focused on discovering insights into sequences of discrete events, with a broad range of practical applications. For example, SPM can be applied to various types of sequential data, including clickstream data of customers (Melnykov, 2016), DNA sequences (Fokianos and Kedem, 2003; Weiß and Göb, 2008), and protein sequences (Krogh et al., 1994). However, analysing these sequences, e.g., for personalised marketing or behaviour analysis, can be challenging, particularly when the number of unique events is high, which leads to a very large number of sequential patterns (Fournier-Viger et al., 2017). This issue is particularly pronounced in real-world scenarios, where the number of unique events, such as clicks, searches, and likes, can range from thousands to hundreds of thousands for a modern website (Liu et al., 2016; Olmezogullari and Aktas, 2022; Su and Chen, 2015). Furthermore, a single event can trigger a cascade of underlying events, including internal transactions, status updates, and notifications to external systems, among others, thereby amplifying the complexity of the analysis. Moreover, since SPM is typically unsupervised and agnostic to the end goal (Gan et al., 2019), many discovered patterns may not be relevant or interpretable in the context of specific business outcomes, such as churn prediction. In such cases, the goal is to identify patterns that are highly specific to churning customers, even if they occur infrequently, as these can serve as early warning signals or triggers for targeted retention strategies. While traditional SPM methods tend to highlight frequent patterns, this can obscure less common but more informative^1^ sequences. Previous works (Adda et al., 2007; Darrab et al., 2024) have shown such effects for rare item mining, where the focus is explicitly on infrequent patterns. Furthermore, in large-scale datasets, even rare patterns can achieve statistical significance due to the sheer number of sequences, which complicates the task of isolating truly meaningful signals from statistically significant, but potentially spurious noise. This highlights the need for goal-driven or supervised approaches that prioritise patterns based on relevance to the outcome of interest, rather than frequency or statistical significance alone. Similar problems arise in analysing process mining graphs, which are directed graphs from discrete event logs (Van Der Aalst, 2016). For instance, interpreting and linking the results of a customer satisfaction survey back to the specific sequences of events responsible for churning customers can be challenging due to the high number of nodes in these graphs (Lamghari, 2022). This problem is even more challenging when analysing graphs visually, owing to the numerous event paths, resulting in situations of cognitive overload, error-prone and time-consuming analyses (Lamghari, 2022; Zimmermann et al., 2024).

One way to overcome these issues and to better understand such complex sequential data is to use explainable Artificial Intelligence (xAI) (Molnar, 2025; Lou et al., 2012). xAI frameworks, such as SHAP (Lundberg and Lee, 2017) or LIME (Ribeiro et al., 2016), offer explanations as to which sequences have the most predictive power. However, the application of such frameworks, in particular deep neural networks for modelling sequential data, is often hindered by computational costs, error susceptibility (Atzmueller et al., 2024; Rudin, 2019; Bilodeau et al., 2024) and potential financial constraints from a scalability and business perspective (Cubric, 2020). As a result, simpler approaches with glass-box models, such as decision trees and generalised additive models, are often preferred (Hastie and Tibshirani, 1987; Rudin, 2019). Yet these models have their own challenges, particularly when dealing with sequential patterns. To evaluate the impact a sequential pattern has on the underlying classification variable, it needs to be isolated as a one-hot encoded feature (Mougan et al., 2023). This process, however, can become quickly unfeasible due to the high-memory requirements of one-hot encoding (Yang et al., 2018; Xiang et al., 2020), which is especially true for a large number of sequential patterns.

Thus, there is a pressing need to reduce the number of sequential patterns in time series data in a way that maintains alignment with a supervised classification goal, while supporting interpretability and scalability. Addressing this gap, our study explores the integration of unsupervised SPM with supervised learning to filter and reduce the number of meaningful patterns before feature encoding. This approach aims to balance interpretability, computational feasibility, and relevance to a downstream classification task.

To this end, we investigate the following research questions:

- **RQ1**: Can unsupervised sequential pattern characteristics be used as a reliable indicator for selecting the most informative patterns that contribute to accurate binary classification?
- **RQ2**: If so, how well does our feature selection criterion compare to existing feature selection algorithms, such as mutual information or feature importance from decision trees?

To investigate our research questions, we quantify the impact a sequential pattern has on a binary classification problem by correlating its confidence measure class-wise, and we are able to reduce the number of sequential patterns through statistical significance tests. To assess the effectiveness of our proposed feature selection process, we conducted a comprehensive evaluation with (1) an artificially generated classification dataset, enabling control of the informativeness of the sequences, and (2) two real-world sequence datasets for malware detection and clickstream analysis (RQ1). For all datasets, our correlation analysis of the delta confidence measure, based on which we select the sequences, shows a statistically significant positive correlation with the target variable. These results imply that our feature criterion can be used to determine the influence that a sequence has on a classification problem. Moreover, we show the importance of the mined sequential patterns by comparing the downstream classification performance on all datasets with existing feature selection algorithms (RQ2). The results indicate that our feature selection criterion performs equally well on two of three datasets compared to existing feature selection algorithms. In terms of computational time and memory usage, we demonstrate that our feature selection algorithm is more efficient than one-hot encoded sequential patterns with the subsequent application of interpretable machine learning methods.

With this new feature selection methodology, we contribute to existing information systems literature by proposing a novel and utility-independent way to use sequential pattern algorithms to mine and rank informative sequential features. The overall principle generally applies to any SPM algorithm and does not require any algorithmic modifications. Using information about the binary target variable during the SPM process and subsequent testing for statistical significance reduces the need for memory-intensive feature selection for downstream classification problems. In comparison to existing methods that rely on statistical association metrics such as the phi coefficient, 1-quality (Pellegrina and Vandin, 2024), Chi-squared, or entropy, our approach offers a locally sensitive measure of pattern impact that is based on class-conditional dependencies. The phi coefficient and 1-quality capture global associations between two binary variables but tend to dilute the importance of class-specific patterns, especially those confined to small subgroups. In contrast, Chi-squared and entropy are more sensitive to local patterns; however, their non-linear behaviour can make the results difficult to interpret. By providing a class-local estimate of impact, our method makes it easier to identify and understand patterns that are specific to particular classes, even when they occur infrequently. This interpretability helps practitioners uncover hidden correlations in their sequential data. For instance, these findings can be used in customer interaction analysis to enhance recommendations, bundling, and offerings. Furthermore, the mined sequential patterns also represent an innovative starting point for feature engineering, with the potential to enrich already existing machine-learning models with novel feature sets.

The remainder of our paper introduces related work in Section 2. In Section 3, we explain our novel feature selection method and the evaluation methodology. In Section 4, we elaborate on our experiments and discuss the results in Section 5. Lastly, we summarise our findings and outline directions for future research in Chapter 6.

## 2 Related work

In the following section, we review the feature selection problem for SPM from various perspectives. First, we introduce the fundamental aspects of SPM problems. Then, we provide an overview of algorithms used to model sequential data, including categorical time series and state-of-the-art deep learning methods. Lastly, we review the most common feature selection methods employed for binary classification problems to form the basis for evaluating our approach.

### 2.1 Association rules and sequential pattern mining

Association rules, introduced in the early 1990s (Agrawal et al., 1993b,a), find relationships and dependencies between items that co-occur in a dataset. Typically, association rules are expressed in the form of *A* → *B*, where A and B represent sets of items. One of the most well-known applications of association rules is market basket analysis, where the goal is to discover relationships between items frequently purchased together. By identifying such associations, businesses can gain insights into customer behaviour and make informed decisions regarding product placement, cross-selling, and promotions. The original algorithm for mining association rules, known as Apriori, was proposed by Agrawal et al. (1993b). It employs a principle known as the Apriori property, which asserts that all subsets of a frequent itemset must also be frequent. However, due to its iterative approach that requires multiple database scans, it was found to be computationally inefficient in practice. A more efficient algorithm called FPGrowth was later introduced by Han et al. (2004). The FPGrowth algorithm efficiently finds frequent itemsets by using a compact prefix tree (FP-tree) to avoid candidate generation, whereas the Apriori algorithm generates and tests candidate itemsets, making FP-Growth faster and more memory-efficient for large datasets.

To evaluate association rules, support and confidence measures as shown in Equations 1, 2 are used. While support captures the frequency of an association rule, confidence describes the conditional probability of a rule, given a certain prior.


(1) $$\begin{array}{l} \text{support}\left(A,\;B\right)\;=\;P\left(A\;|\;B\right) \end{array}$$

(2) $$\begin{array}{l} \text{confidence}\left(A\to B\right)=P\left(B|A\right) \end{array}$$

While support and confidence are commonly employed to evaluate association rules, these metrics have notable limitations. In particular, they overlook statistical correlations and may fail to reflect genuine dependencies between events (Morishita and Sese, 2000; Sese and Morishita, 2002; Llinares-López et al., 2015). To address this, Piatetsky-Shapiro (1991) propose the use of lift, defined in Equation 3.


(3) $$\begin{array}{l} \text{lift}\left(A,B\right)=\frac{P\left(A,B\right)}{P\left(A\right)P\left(B\right)} \end{array}$$

Although lift is useful for identifying deviations from independence, it can still yield association rules that are statistically insignificant (Hämäläinen and Nykänen, 2008). To address this limitation, alternative interestingness measures based on statistical hypothesis testing have been proposed to more rigorously assess the significance of associations (Webb, 2006). These methods typically rely on a 2 × 2 contingency table, as shown in Table 1, which summarises the joint and marginal frequencies of itemset occurrences and non-occurrences. This table serves as the foundation for statistical tests such as the Chi-squared test and Fisher's exact test, both of which evaluate whether item co-occurrence significantly deviates from what would be expected by chance. Like the Chi-squared statistic, the 1-quality measure, which is also known as leverage, can be used as an alternative to assess the association between a pattern and the target variable (Pellegrina and Vandin, 2024). This measure captures how often a pattern and the target variable co-occur, compared to what would be expected if they were independent. In other words, it quantifies the gap between the observed frequency of a pattern appearing in transactions labelled with 1 and the frequency we would expect under the assumption that the pattern and the target are unrelated.

**Table 1:** 2 × 2 contingency table showing the joint distribution of itemsets *A* and *B*, with *a*, *b*, *c* and *d* being the corresponding absolute frequencies.

**Itemset**	***B***	** $\bar{B}$ **	**Total**
*A*	*a*	*b*	*a* + *b*
$\bar{A}$	*c*	*d*	*c* + *d*
Total	*a* + *c*	*b* + *d*	*n*

Algorithms such as AprioriSMP (Morishita and Sese, 2000) and TidalSMP (Sese and Morishita, 2002) incorporate the application of these tests, employing horizontal and vertical mining strategies utilising p-values to identify statistically significant itemsets. To combat the multiple testing problem in significant pattern mining,^2^ which searches for the most significant patterns, one can control for false discoveries (e.g., False Discovery Rate (FDR) (Benjamini and Hochberg, 1995; Benjamini and Yekutieli, 2001) or Family-Wise Error Rate (FWER) (Bonferroni, 1936; Holm, 1979)). In their tutorial, Pellegrina et al. (2019) provide a comprehensive overview of existing methods. For instance, Terada et al. (2013a) introduced the “Limitless Arity Multiple-testing Procedure” (LAMP), which enhances efficiency by pruning itemsets based on their minimum attainable p-values. The computational performance of LAMP was further improved in subsequent work by Minato et al. (2014). Alternatively, Terada et al. (2013b); Llinares-López et al. (2015) applied permutation tests to adjust for multiple comparisons.

Despite their effectiveness in discovering associations, the aforementioned approaches do not consider temporal constraints in their rule mining process. In other words, they only look at the frequency of items but ignore the order of their occurrence. To address this limitation, SPM algorithms have been developed. SPM algorithms can be divided into two categories, namely Apriori-based and pattern-growth approaches (Millham et al., 2021). Apriori-based algorithms, such as Sequential PAttern Discovery using Equivalence classes algorithm (SPADE) (Zaki, 2001) and Generalised Sequential Pattern algorithm (GSP) (Srikant and Agrawal, 1996), generate a large number of sequence candidates, which are then tested for a specified minimum support threshold. Pattern-growth algorithms, such as PrefixSpan (Pei, 2001) and FreeSpan (Han et al., 2000), solve the computational issues of large candidate generation by introducing an efficient search space partitioning (Abdullah et al., 2019).

While these algorithms provide a computationally efficient way of mining sequential patterns, they typically result in a large set of sequences that are often redundant and do not necessarily carry information about the underlying classification problem (Gan et al., 2019). One way to minimise redundancy is to use Closed and maximal sequential patterns. A closed sequential pattern (Han et al., 2013) retains frequency information by ensuring no supersequence has the same support, while a maximal sequential pattern ensures no supersequence is frequent, keeping only the longest relevant patterns (Fournier-Viger et al., 2013). However, these approaches fall short in accounting for correlation with external classes since statistical measures such as Chi-squared or the correlation coefficient are anti-monotone (Morishita and Sese, 2000; Sese and Morishita, 2002). In this context, non-maximal or open patterns might still hold high explanatory value. Another solution is high-utility SPM, which considers the utility of each sequential pattern during the mining process (Truong-Chi and Fournier-Viger, 2019). For utility-based pattern mining, its associated algorithms, such as USpan (Yin et al., 2012) and CHUSP (Dinh et al., 2023), generally assume that a utility value is specified for each event in the sequence. However, in a classification setting, such utility measures are not explicitly available, which makes the algorithms unsuitable. Similar to the previous work on statistical testing for itemsets, Dalleiger and Vreeken (2022) also leverage an upper bound during the mining process to increase the efficiency, while Tonon and Vandin (2019) proposes using the Westfall-Young method for multiple hypothesis testing with SPM. Our work complements these existing works on statistical testing by proposing a more streamlined approach that repurposes existing SPM algorithms without in-built statistical testing capabilities to compute a novel measure of interest. This novel measure captures the directional and bounded discriminative effect size that a pattern exhibits with respect to a given binary classification problem and is based on conditional, within-group differences.

### 2.2 Categorical time series modelling

A binary time series classification problem involves categorising sequences of time-dependent data (time series) into one of two distinct classes (Lin et al., 2015). Each time series consists of ordered data points collected over time, and the goal is to train a model to predict whether a given time series belongs to one of the two predefined classes, typically based on patterns or trends in the temporal data (Ismail Fawaz et al., 2019). We refer to this time series as categorical when dealing with discrete data points as features.

There are two ways of modelling a binary time series classification problem: (1) using time series models directly or (2) extracting features from the time series and treating them as a tabular and time-invariant dataset as input to a regression problem (Fulcher and Jones, 2014). Compared to numerical time series, categorical time series require learning a numerical representation of the categorical values. One of the earliest approaches to model categorical time series is Markov chains (Gagniuc, 2017), which use a transition matrix to estimate the next event. Lin et al. (2022) introduces a hidden Markov ensemble algorithm that uses the Wasserstein distance and autoencoders to learn discrete features of time series, combined with a hidden Markov model for learning continuous features. An alternative approach is given by Wang et al. (2021), who propose an end-to-end representation learning model for time series classification, utilising temporal convolution, residual networks, bidirectional long short-term memory (LSTM) networks, and a multi-layer perception network. Similarly, the Temporal Fusion Transformer (Lim et al., 2021) proposes a neural network architecture that not only learns a representation of a time series but also combines it with static, time-independent features to solve a classification problem. However, such neural network based architectures are considered black-box models. To interpret such them on an observation level, we need an additional interpretability component, such as SHAP (Lundberg and Lee, 2017) or one of its time-aware derivatives (Nayebi et al., 2023; Raykar et al., 2023). This, however, adds more complexity and runtime, necessitating more straightforward approaches in practice.

An easier-to-interpret and computationally less demanding way of determining significant features for a classification problem is through machine learning, where the importance of features can be learned based on their correlation to the underlying problem (Saarela and Jauhiainen, 2021; Liu et al., 2022). However, the features must be mined manually beforehand, involving domain knowledge and feature engineering to create meaningful features (Dong and Liu, 2018). In the case of numerical time series, such features are overall trends, seasonality, stationarity, lagged values and other measures of central tendency, such as the minimum or maximum value (Mukhopadhyay and Samanta, 2023). A more advanced technique of mining features is time series shapelets, which aims at finding the most representative numerical time series subsequence for a given class (Ye and Keogh, 2009). However, these numerical features do not apply to a categorical time series since categorical values cannot be directly transformed to an ordinal scale (Lin et al., 2015; Fokianos and Kedem, 2003). Instead, one common technique used to encode categorical events is binary encoding, also known as one-hot-encoding (Suits, 1957). Although this technique disregards the temporal relationships within sequences, it identifies whether a specific sequence appears in an observation. As a result, it is commonly used in interpretable machine learning (Davis, 2021; Alkharusi, 2012). However, we obtain a memory-intensive feature matrix by doing so, making it challenging to apply this approach in practice (Xiang et al., 2020). In this study, we propose a memory-efficient method for extracting important sequential patterns from categorical time series data and quantifying their influence on the classification task, thereby making it well-suited for exploratory data analysis and feature selection.

### 2.3 Feature selection algorithms

Feature selection aims to reduce dimensionality in machine learning problems. In their survey, Preyanka Lakshme and Kumar (2022) divide the feature selection process into unsupervised and supervised problems. For supervised problems, the authors further distinguish between (1) filter, (2) wrapper, (3) embedding, and (4) hybrid methods. The filter approach describes the selection of features based on statistical properties, such as the missing value ratio, the correlation coefficient, or the permutation feature importance, ANOVA, Mutual information (Kraskov et al., 2004). Wrapper methods iteratively check if a machine learning model has improved its prediction capabilities due to the inclusion or removal of features. Typical examples encompass the forward (Whitney, 1971) and backward selection (Marill and Green, 1963) of features, as well as genetic algorithms (Leardi, 1996). The embedded and hybrid approaches are mixtures of the already mentioned types. Embedded methods use the filter and wrapper methods within the actual prediction model, implementing their own feature selection during the training process of the machine learning model. A typical example is the L1 or L2 regularisation of linear regression models (Ng, 2004). Hybrid approaches, in contrast, use a combination of filter and wrapping methods. A prominent example of such a hybrid system is Boruta (Kursa et al., 2010), which iteratively checks if each feature is more important than randomly shuffled features from the supplied dataset. Based on statistical significance tests, the features are then either retained or discarded. Another advanced hybrid feature selection algorithm is the minimum Redundancy Maximum Relevancy (mRMR) principle (Peng et al., 2005). The idea is to account for redundancy among the important features and thereby maximise the overall discriminative power of the selected features. While the above-mentioned feature selection methods work well with tabular data, they do not consider temporal dependencies of sequential events by design. Hence, to utilise these feature selection algorithms, it is necessary to extract temporal features beforehand. With our work, we address this shortcoming and directly integrate the feature selection into the SPM process. The subsequent section presents a comprehensive delineation of the proposed methodology utilised for the extraction and subsequent selection of temporal features.

## 3 Materials and methods

In this section, we first explain our feature selection algorithm in detail. This includes the criteria used to evaluate the importance of each sequence and the subsequent evaluation (i.e. statistical measure) for assessing the algorithm. The pseudo-code in Algorithm 1 introduces our framework more formally. Next, we describe the datasets we used in our experiments to evaluate the feature selection method and the selection procedure of these datasets. The entire codebase to reproduce the experiments is available at https://github.com/alexandergrote/cts.

**Algorithm 1:** Pseudocode of feature selection process of binary classification problem.

**Require:** Number of bootstrap rounds *Z*
**Require:** Minimum support threshold θ_supp_
**Require:** Maximum sequence length θ_l_
**Require:** Minimum effect size θ_δ_
**Require:** Significance level α
1: **for** *z* = 1 to *Z* **do**
2: Draw bootstrapped sample *s*
3: Apply SPM class-wise with θ_supp_ and θ_l_
4: Calculate δ_*s, r*_ = *P*_*s, r*_(*B*|*A, D*_*pos*_) − *Ps, r*(*B*|*A, D*_*neg*_) for each sequential pattern *r*
5: **end for**
6: Shrink sequences to a unique subset of sequences, ignoring antecedents and precedents
7: **for** each unique sequence *r* **do**
8: Conduct a Mann-Whitney-U test with the alternative hypothesis |δ_*r*_| > θ_δ_
9: Correct for multitesting
10: Keep sequence based on corrected p-value < α
11: **end for**

### 3.1 Feature selection algorithm

The main idea of the feature selection algorithm is to first capture sequential patterns for each class of the binary classification problem separately. Next, we calculate the difference in the confidence measure ∈[0, 1] for each pattern *r* and select the most important sequences based on this difference.


(4) $$\begin{array}{l} \delta_{r}=P_{r}\left(B|A,D_{pos}\right)-P_{r}\left(B|A,D_{neg}\right) \end{array}$$

Equation 4 illustrates the underlying concept formally, where *D*_*pos*_ represents the set of positive examples and *D*_*neg*_ the negative examples. For each sequence *r*, we calculate a difference δ_*r*_ yielding values within the range [-1,1]. This difference represents the contrast in confidence levels between two subsets: one containing only positive class values and the other containing only negative class values. The absolute value of δ_*r*_ serves as an indicator of the sequence's impact on classification. A value close to 1 indicates a strong influence, while 0 suggests the sequence is equally influential for both classes. Furthermore, the sign of δ_*r*_ provides additional insight: negative values indicate a greater impact on the negative class, whereas positive values indicate a greater impact on the positive class. In this work, we used the PrefixSpan algorithm (Pei, 2001) to mine sequential patterns, but the overall procedure is independent of the SPM algorithm.

We repeat the overall process *Z* = 10 times with stratified, random sampling to better reflect the underlying aleatoric uncertainty. This means each fold contains the same proportions of classes, but the data points are drawn at random. The choice of *Z* is arbitrary; however, we select ten as a balance between the computational cost of SPM and predictive accuracy. This choice aligns with findings in cross-validation research, suggesting that 5-10 folds is generally sufficient for model evaluation (Kohavi, 1995; Breiman and Spector, 1992; Hastie et al., 2009). We store the confidence difference between positive and negative classes for each run. As an initial measure to diminish the number of sequences, we employ two minimum support thresholds: an absolute threshold of 100 occurrences, which guarantees the existence of a sufficient number of data points for statistical analysis, thereby precluding the extraction of numerous unimportant patterns. Afterwards, we shrink the number of sequences by only considering unique ones. This is important since sequences may have different antecedents and consequents but share the same sequence of events. For instance, the sequence *A* → *B* → *C* consists of two sequential patterns with different confidence estimations: *P*(*C*|*A* → *B*) and *P*(*B* → *C*|*A*). In our case, we have retained the sequences with the highest absolute delta confidence measure and disregarded the remaining sequences.

To select discriminative features that have statistically significant effects on the classification problem, we conduct a Mann-Whitney-U (MWU) test (Mann and Whitney, 1947; Wilcoxon, 1945) on the confidence differences for each sequence. This non-parametric approach was selected based on several methodological considerations. First, the data exhibited non-normal distribution patterns and were measured on an ordinal scale, while also meeting the critical assumption of independence between comparison groups. Additionally, the dataset comprised absolute delta confidence values strictly bounded between 0 and 1, which precluded the use of parametric tests that assume unbounded, normally distributed continuous data. The number of samples in this context depends heavily on the number of bootstrap iterations, which directly influences the stability and reliability of the resampled estimates. Finally, the MWU test offers robust performance with small sample sizes and is relatively unaffected by outliers, an advantage given the constrained range and potential skewness of our measures. In particular, we are interested in sequences with an absolute delta confidence value greater than 0. To further reduce the chances that a sequence is considered important by randomness and to ensure a sufficiently large effect size for practical significance, we require the absolute delta confidence value of the sequences to be above a user-defined delta confidence threshold. To combat the multitesting problem of inferring only based on observed values and to control the false discovery rate (i.e., identify as many significant features as possible while incurring a relatively low proportion of false positives), we adjust the *p*-values via Benjamini-Yekutieli correction (Benjamini and Yekutieli, 2001). Unless stated otherwise, we have considered the maximum sequence length of 3 as a length constraint, similar to prior work on website/clickstream data based on online retail behaviour (Desai and Ganatra, 2015). Longer sequences generally have lower support and are unnecessary to demonstrate our feature selection algorithm.

### 3.2 Datasets

In this section, we will introduce the three datasets with sequential patterns, based on which we conducted our evaluation. We primarily use a synthetic dataset to provide a controlled environment and showcase the inner workings, and then utilise two real-world datasets to test the applicability of the proposed algorithm in practice. In particular, we utilise a dataset on malware detection through API call sequences (Oliveira, 2019) and a dataset on customer churn with clickstream data Requena et al., (2020). After a brief description of each dataset and its preprocessing, we compare and provide an overview of the statistical properties of each dataset.

The synthetic dataset consists of 20,000 sequences composed of 15 unique events, which we will denote as separate letters, ranging from “A” to “O” in the Latin alphabet. To generate a sequence, we randomly draw the letters sequentially until we meet the desired sequence length, which we also select randomly to be between 2 and 15. We control for the informativeness by imposing mutually exclusive constraints, that is, we require some sequences to be only indicative for one class and not to appear together with another informative sequence. In our case, *A* → *B*, *B* → *C*, *C* → *D*,*D* → *E* are indicative for the positive class and each of the subsequences occurs in 10 % of all sequences. Likewise, *F* → *G*, *G* → *H*, *H* → *I*, *I* → *J* signal the negative class, and each subsequence also occurs in 10 % of all sequences. With these sequences, we can predict the classes of 80 % of all sequences, and the classification results of the remaining 20 % are subject to chance.

The malware dataset consists of 44,058 API call sequences resulting from a 3,000-hour-long execution of malware and goodware data points in a Cuckoo Sandbox environment (Oliveira, 2019). It consists of 43,979 malware and 1,079 goodware call sequences, constituting the two classes in this classification task. Each sequence has 100 non-consecutive API calls encoded as integer values, which means the same API call does not occur in direct succession. To avoid class imbalance effects, we downsample the malware class at random.

The raw customer churn clickstream data stems from a fashion e-commerce website over two months in 2018 Requena et al., (2020). It contains 443,652 anonymised sessions of clickstream trajectories of website visitors. Each session represents a series of events that occur within 30 min. The six unique clickstream events, which are “Page view”, “Detail”, “Add”, “Remove”, “Purchase”, and “Click”, describe actions on the website. Notably, only 2.08 % of these clickstream sessions culminate in purchases, thereby rendering it a profoundly imbalanced classification problem. To maintain comparability, we conduct the same preprocessing on the raw data as (Requena et al. 2020). This implies that we keep sequences that are longer than 4 clicks to ensure that the sequence contains enough events for classification, and we trim the sequences by only considering the clicks that occurred before a purchasing decision. Furthermore, we also downsample the majority class to create a balanced classification problem and remove sequences that are unreasonably long for a 30-minute session. Just as (Requena et al. 2020), we choose 155 as the maximum allowed sequence length, equalling a reduction in observations of only 1 %.

Table 2 illustrates key characteristics of the resulting preprocessed datasets. While they all yield a balanced class ratio, the number of unique events and sequence lengths differ significantly. The malware dataset has, on average, the longest sequence length and the highest number of unique events. The synthetic dataset, on the other hand, has the shortest sequences on average, and the churn dataset possesses the fewest unique events.

**Table 2:** Statistical properties of preprocessed and downsampled datasets.

**Dataset**	**Number of**		**Sequence length**		
	**Sequences**	**Unique events**	**Min**	**Average**	**Max**
Synthetic	15	20,000	2	7.14	14
Malware	232	2,158	100	100	100
Churn	4	11,948	5	20.76	155

## 4 Results

We conduct a comprehensive evaluation of our proposed algorithm through six distinct approaches. Firstly, we examine the correlation between the delta confidence measure and the target variable, as presented in Subsection 4.1. Secondly, we compare the delta confidence with existing interesting measures in Subsection 4.2, followed by an ablation study of the effect of different hyperparameters on runtime, number of selected sequences and classification accuracy in Subsection 4.3. Next, we compare the efficacy of our proposed solution with existing feature selection algorithms in Subsection 4.4. This is succeeded by a benchmark analysis in Subsection 4.5, which contextualises the obtained classification accuracies in relation to a fine-tuned classifier. Finally, we conclude the chapter with Subsection 4.6, wherein we present a cost-benefit analysis of our algorithm, in comparison to other feature selection algorithms.

### 4.1 Feature selection criterion analysis

To analyse the individual steps of our proposed feature selection algorithm, we track the number of sequences remaining after each step in Table 3. For clarity in the discussion and analysis, we refer to the aggregated mined sequences after bootstrapping. Notably, applying SPM on the malware dataset results in a very high number of sequences due to 1) a high number of unique events and 2) a sequence length of 100 for each observation.

**Table 3:** Number of sequences after each selection step.

**Steps**	**Dataset**		
	**Synthetic**	**Malware**	**Churn**
Unique patterns	210 (100 %)	51,747 (100 %)	119 (100 %)
Aggregated patterns	209 (100 %)	26,751 (52 %)	67 (56 %)
Statistically significant patterns	107 (51 %)	23,161 (45 %)	54 (45 %)

**Figure 1:**
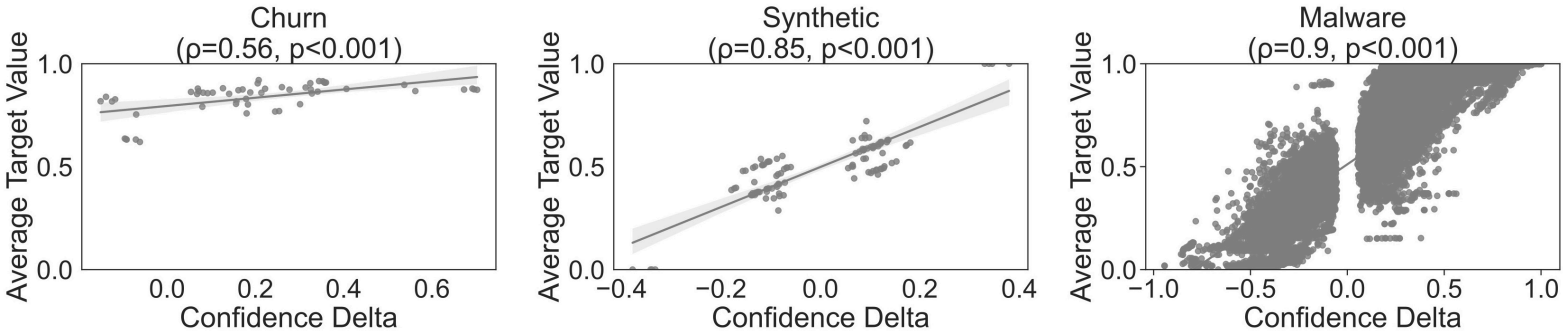
Feature selection criterion analysis.

Figure 1 demonstrates the correlation between the target variable and the delta confidence measure (RQ1). We observe a statistically significant positive linear correlation, as measured by the Pearson correlation coefficient ρ, for all three datasets, albeit to varying extents. The synthetic and malware datasets exhibit an almost perfect linear correlation of 0.85 and 0.9, while the churn dataset only yields a correlation coefficient of 0.56. Furthermore, as expected, no sequences with a delta confidence around δ = 0 exist. Instead, sequences with δ> 0 have, on average, a higher number of positive observations, whereas the reverse is true for sequences with δ < 0. In the synthetic dataset, the extreme points are represented by their weighted sequences, which also matches our expectations.

### 4.2 Comparison to alternative statistical measures

**Figure 2:**
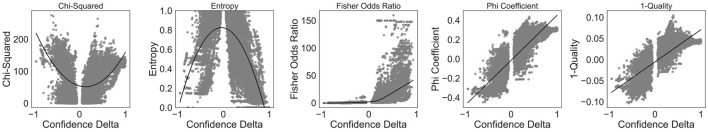
Subgroup Interesting measure analysis.

As noted by Geng and Hamilton (2006) and Tan et al. (2004), a multitude of interesting measures exist. In this section, we compare the delta confidence criterion against five of the most prevalent measures, namely Chi-squared ∈[0, ∞], entropy ∈[0, 1], the Fisher odds ratio ∈[0, ∞], the Phi-statistic ∈[−1, 1] and the 1-quality ∈[−1, 1]. The previous visualisation of delta confidence values in Figure 1 reveals that only the malware dataset exhibits delta confidence values ranging from -1 to 1. As a consequence, this is the only dataset that can give a complete overview of the relationship between other interesting measures and the delta confidence criterion, which Figure 2 visualises.^3^ For the Chi-squared and entropy, we observe a parabolic trend, whereas for the Fisher Odds Ratio, we see an exponential correlation. Given the symmetric nature of Chi-squared values and entropy, it is not possible to deduce the influence direction from these metrics alone. The Fisher odds ratio ranges from ∈[0, ∞], whereby a value < 1 indicates a negative influence and a value >1 a positive one. However, given the asymmetric nature of the importance curve, these interestingness measures are difficult to interpret.

A particular interesting comparison is between the delta confidence, the phi coefficient and the 1-quality criterion. While the theoretical bounds of Phi (±1) and 1-quality (±1) are seldom reached in practice, delta confidence more readily approaches its extremes when patterns are highly class-specific. This disparity arises from their distinct methodological underpinnings: delta confidence quantifies directional association by normalising the difference in pattern occurrence across classes, whereas the Phi-statistic is derived from the full confusion matrix, considering both the presence and absence of a pattern. Similarly, the 1-quality measure, derived from statistical independence, relies on joint and marginal probabilities from the contingency table, often yielding smaller values. Hence, both the Phi coefficient and 1-quality provide a more conservative, global assessment of association, reflecting the influence of cases where the sequence does not occur. In contrast, delta confidence highlights local, class-specific associations.

This difference has practical implications, especially in applied domains like e-commerce or fraud detection. Consider a behavioural sequence, e.g., [newsletter → product → checkout], that occurs almost exclusively among premium users. Even if rare in the total dataset, delta confidence will correctly yield a value close to +1, highlighting the pattern's strong class specificity. The phi coefficient or 1-quality measure, on the other hand, will under-represent the sequence's discriminative power due to the dilution from observations that do not comply with the sequence. From a business perspective, such high delta confidence patterns are valuable for tasks like targeted marketing or early customer profiling, where the goal is to identify precise, class-specific signals rather than optimise global prediction performance.

**Figure 3:**
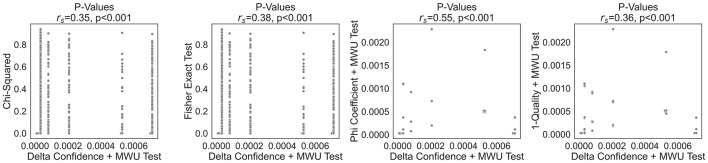
*P*-values comparison.

Beyond correlation analysis, further insights emerge when considering p-values, which are illustrated in Figure 3. The figure compares the p-values obtained using the delta confidence combined with the MWU test to those derived from the Chi-squared test and Fisher's exact test. It also includes a comparison with a similar setup to the delta confidence + MWU test, but instead of using delta confidence, it employs the phi coefficient along with the 1-quality criterion. Notably, for a fixed p-value obtained through the delta confidence + MWU approach, we observe differing *p*-value magnitudes for the Chi-squared and Fisher's exact test. For the alternative setups with the phi coefficient and 1-quality, we cannot observe such a clear pattern. This suggests that, despite their monotonic correlation as indicated by the Spearman coefficient *r*_*s*_, the delta confidence and conventional subgroup interesting measures may carry different information and therefore complement each other. While delta confidence measures conditional, within-group differences, the subgroup interesting measures assess the significance of observed patterns based on frequency distributions. Importantly, even when the MWU test confirms the statistical significance of the delta confidence values, the Chi-squared or Fisher's exact test can yield different results, due to inherent differences in the data and hypothesis (such as continuous vs. categorical data, or testing for differences in distributions vs. testing for independence). In summary, delta confidence offers directional, subgroup-sensitive properties that are particularly useful for decision-making scenarios that require understanding nuanced behaviours within specific subpopulations.

### 4.3 Ablation study on hyperparameters

**Figure 4:**
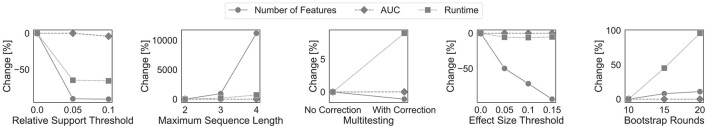
Ablation study on hyperparameters.

To elucidate the impact of the hyperparameters on our proposed feature selection process, we conducted ablation studies, controlling for accuracy, number of remaining sequences, and overall runtime on the synthetic dataset. Figure 4 synthesises the effects of the minimum support, maximum sequence length, multitesting, minimum effect size, and the number of bootstrapping rounds. Overall, each threshold value was subjected to five iterations, and for enhanced readability, we report solely the average values of the results. To better show the effect of multiple bootstrap rounds and the maximum sequence length, we have set the minimum support threshold for these ablation studies to 0. As anticipated, the minimum support precipitously reduces the overall runtime and sequence count until no sequences surpass this threshold. The overall classification accuracy remains unaffected. The maximum sequence length also shows the expected effects: for an increasing maximum sequence length, the runtime increases exponentially while the AUC values and the number of significant features do not change significantly. We can also observe that multitesting reduces the overall number of sequences, has no effect on the overall classification accuracy, but introduces a computational overhead in terms of runtime. Elevating the minimum effect size yields a drastic reduction in the overall feature count and runtime, while accuracy is maintained until no sequences are available anymore. With regard to bootstrapping, it is unsurprising that the runtime increases linearly with increasing bootstrap rounds, while overall classification performance remains largely unchanged. However, with fewer bootstrap rounds, the statistical power of the MWU test is low, which can result in no sequences passing the significance test. In contrast, higher numbers of bootstrap rounds increase statistical power, leading to more sequences reaching significance and a more stable delta confidence distribution.

### 4.4 Feature selection comparison

**Figure 5:**
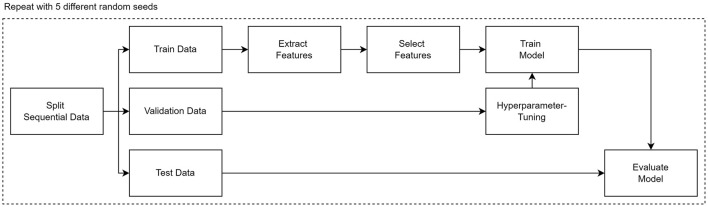
Machine Learning workflow for experiments.

To address RQ2, we conduct a comparative analysis of the feature selection capabilities of the delta confidence measure with respect to existing algorithms. However, instead of directly using the absolute delta confidence measure, we create a new ranking ∈ [0,1] by multiplying the relative support values with the absolute delta confidence values. In this way, we also account for the frequency of each pattern, which is independent of the delta confidence measure. We adhere to a machine-learning workflow as illustrated in Figure 5, partitioning our data into training and testing sets using a stratified 80:20 split. Furthermore, for increased robustness, we repeat each experiment five times with different random seeds each time and employ three well-established algorithms: Naïve Bayes, Logistic Regression, and an eXtreme Gradient Boosting (XGB) (Chen and Guestrin, 2016) classifier. Before training and evaluating each classifier on the area under the curve (AUC) of the receiver operating characteristic (ROC), we utilise a feature selection algorithm to select the most informative features based on the training data. For benchmarking purposes, we deliberately select three established feature selection methods that each belong to a different category introduced in Subsection 2.3: mutual information (filter), random forest feature importance (embedding), and an adaptation of mRMR (hybrid). Furthermore, we have also considered the Chi-squared as an additional filter method to reflect statistical significance testing. By selecting at least one representative from each category, we aim to achieve a broad diversification of different feature selection methods, allowing for a comprehensive comparison with the delta confidence measure. We exclude the wrapper category from consideration due to the high computational costs associated with its iterative process. In terms of the employed mRMR adaptation, we base our mRMR feature selection on the feature importance of a random forest, which was first introduced by Zhao et al. (2019). To control for the redundancy (i.e. high correlation), we use Theil's U ∈ [0,1], an asymmetric correlation measure between categorical variables. In addition to comparing our approach with existing feature selection methods, we conduct a separate analysis on the impact of sequence encoding techniques. Specifically, we contrast one-hot encoded events with one-hot encoded sequential patterns to elucidate the overall significance of sequential patterns in classification tasks. For better readability, we report only the mean AUC values.

**Figure 6:**
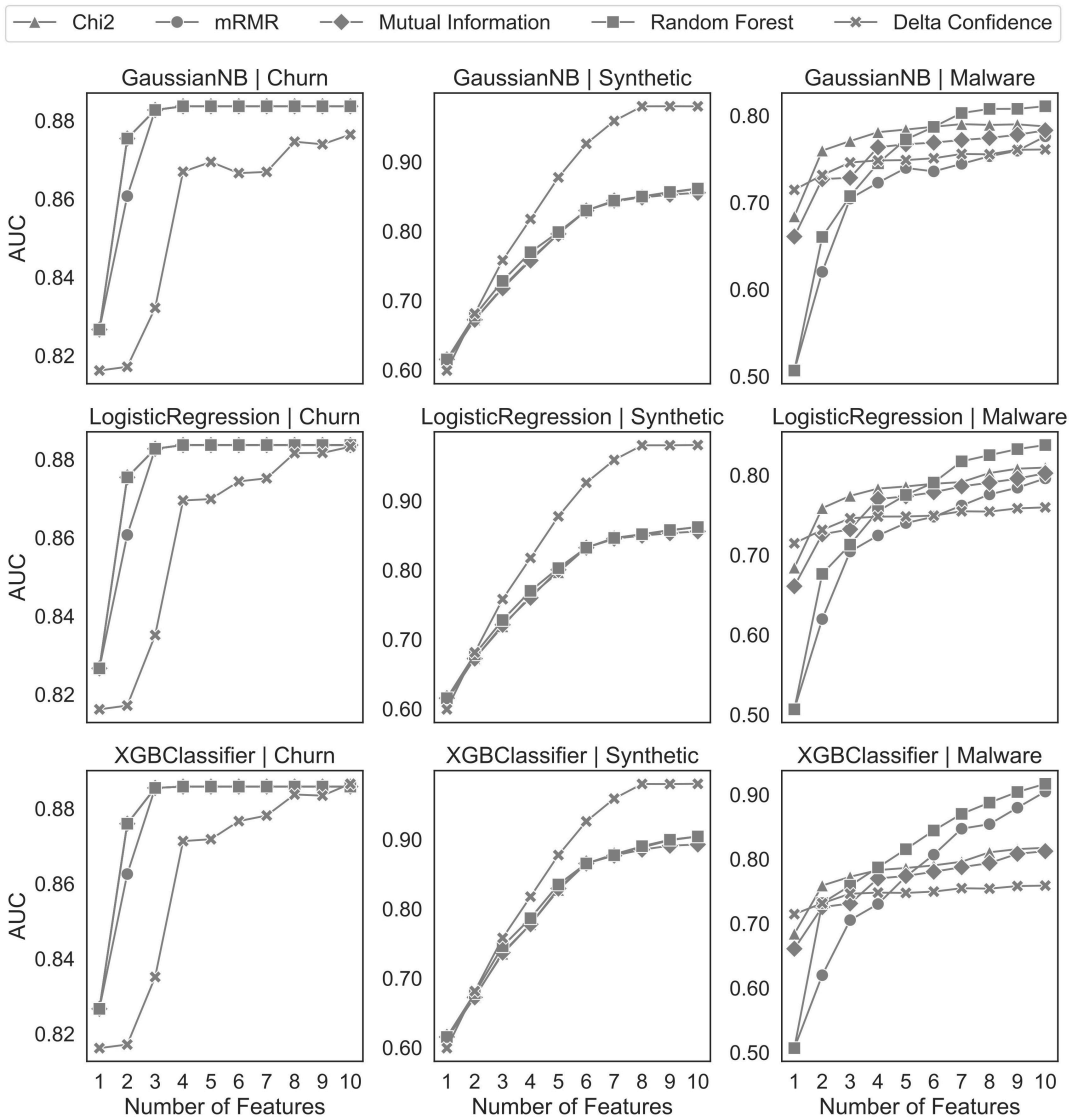
Feature selection analysis on one-hot encoded events.

Figure 6 shows that the overall effectiveness of sequential patterns versus one-hot encoded events depends on the characteristics of the datasets. The feature selection based on one-hot encoded events for the churn dataset shows better results than our proposed sequential pattern feature selection for all benchmark algorithms. The AUC values on the synthetic dataset indicate the opposite result. On the malware dataset, the sequential patterns initially perform better than the one-hot encoded events, indicating that there is one pattern which is particularly important for the classification problem. However, with more features, the results plateau and the event-based feature selection methods achieve better results.

**Figure 7:**
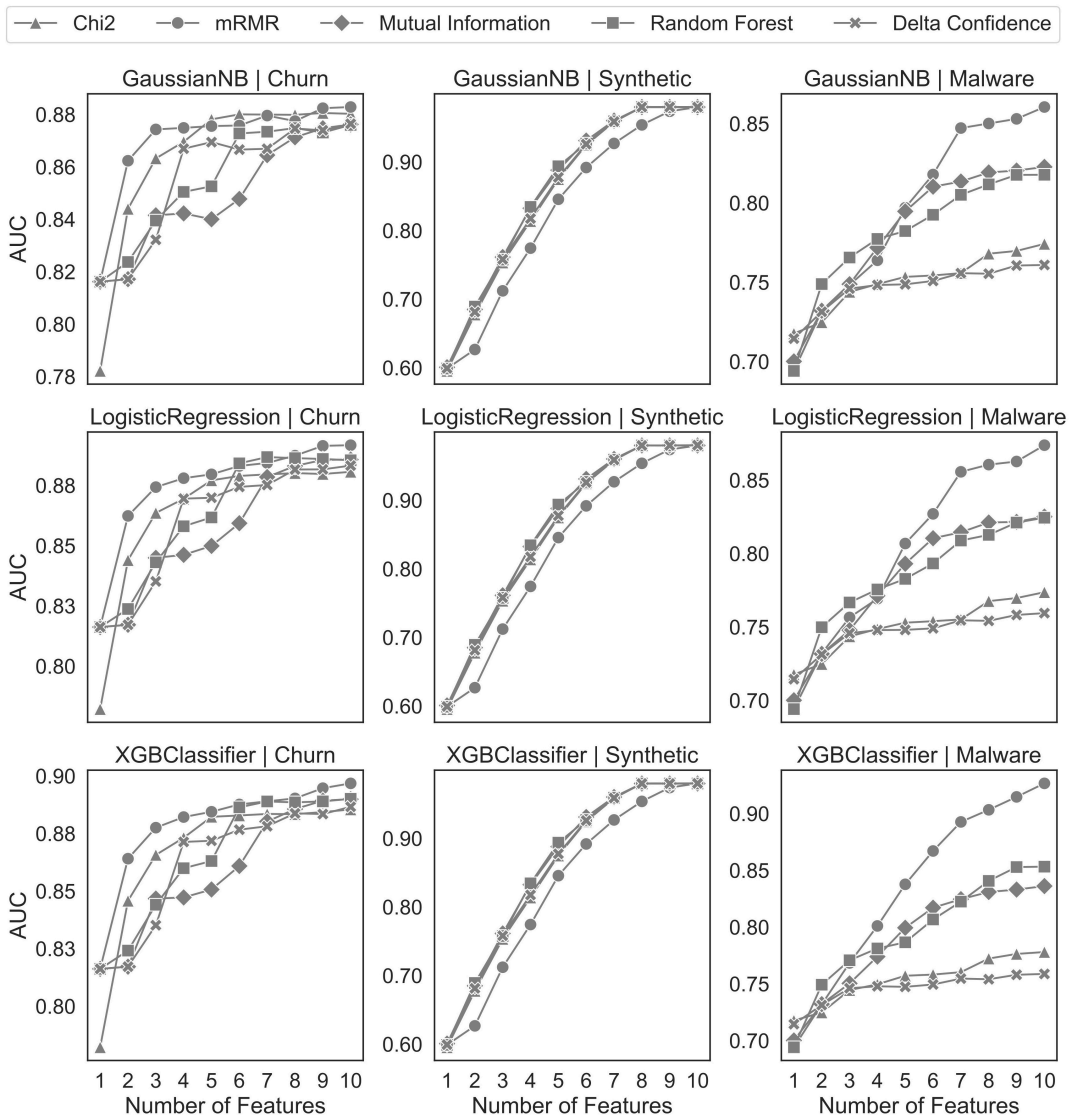
Feature selection analysis on one-hot encoded sequential patterns.

In Figure 7, we benchmark the delta confidence measure with the other feature selection algorithms on one-hot encoded sequential data. For a fair comparison, we use the same preprocessing as our proposed algorithm but different feature selection algorithms after discarding the uninformative sequences by the statistical tests. Our proposed solution achieves results comparable to those of the churn and synthetic datasets of the benchmark feature selection algorithms. However, the feature selection with the delta confidence-based ranking on the malware dataset performs similarly to the Chi-squared feature selection but worse than the other algorithms.

### 4.5 Benchmark study

As an additional analysis to investigate the robustness of overall sequential patterns (RQ2), we assess their importance in our study and contextualise the classification results from the feature selection analysis. Specifically, we compare the performance of various encoding schemes and machine learning models to evaluate the consistency and impact of sequential patterns across different methodological approaches.

In the following, we describe the experimental setup for these scenarios. We use a XGB classifier for the first two cases, and a LSTM model for the third case. For all scenarios, we follow the machine-learning workflow outlined in Section 4.4, with the addition of hyperparameter tuning for the employed models. We use a shallow LSTM model, consisting of three layers, as a proxy for a more complex network. Although we recognise that deeper architectures often produce better results, we opted for a simpler model due to the risk of overfitting for the given sample sizes. The first layer of the employed architecture is an embedding layer that transforms the discrete sequences into a dense representation. Next, the representation is passed to an LSTM layer with a hidden size of *n*, which is subject to hyperparameter tuning. The final layer is a fully connected layer, which transforms the output of size *n* to the desired binary format. To avoid overfitting, we additionally apply Dropout (Srivastava et al., 2014) to the fully connected layer with a dropout rate of 20 %. For updating the weights during training, we utilise the Adam optimiser (Kingma and Ba, 2014) in combination with binary cross-entropy loss and train for 100 epochs unless the result has not improved for ten consecutive rounds. Table 4 provides an overview of the possible hyperparameters, which have been selected for each scenario by the Tree-Parzen-Algorithm (Bergstra et al., 2011).

**Table 4:** Overview hyperparameters.

**Model**	**Hyperparameter space**		**Hyperparameter results**		
	**Name**	**Possible values**	**Synthetic**	**Malware**	**Churn**
XGB (events)	Number of trees	[1, 100]	43	39	73
	Maximum depth	[3, 10]	4	9	10
	Learning rate	[0, 1]	0.5447	0.8500	0.6894
XGB (sequences)	Number of trees	[1, 100]	51	44	50
	Maximum depth	[3, 10]	8	4	3
	Learning rate	[0, 1]	0.8447	0.7226	0.4441
LSTM	Batch size	16, 32, 64, 128, 256	32	64	256
	Learning rate	[0, 1]	0.0082	0.0014	0.0067
	Hidden Size	16, 32, 64, 128, 256, 512	512	16	128

Table 5 shows the mean and standard deviation of F1 and AUC for five experimental runs with different random seeds, for the above three scenarios. The best values are highlighted in bold. The results show that the performance of the selected sequential patterns, which have subsequently been passed to an XGB classifier, achieve the same or greater classification performance than the one-hot encoded events, which have also been passed to an XGB classifier. The classification accuracy of the LSTM is lower than that of the previously mentioned XGB classifier. The exception is the synthetic dataset, where the LSTM achieves on-par results with the sequentially encoded features.

**Table 5:** Benchmark analysis.

**Dataset**	**Metrics**	**One-hot-encoded events**	**LSTM**	**One-hot-encoded sequential patterns**
Synthetic	F1 Score	0.8045 ± 0.0063	0.8958 ± 0.0087	**0.9044** **±0.0063**
	AUC	0.9069 ± 0.0045	0.9778 ± 0.0016	**0.9794** **±0.0017**
Malware	F1 Score	0,9230 ± 0.0192	0.8960 ± 0.0142	**0.9320** **±0.0180**
Churn	AUC	0.9755 ± 0.0098	0.9503 ± 0.0099	**0.9769** **±0.0053**
	F1 Score	**0.8581** **±0.0052**	0.8536 ± 0.0038	0.8533 ± 0.0114
	AUC	0.8867 ± 0.0052	**0.9137** **±0.0065**	0.9055 ± 0.0056

Bold values indicate the best performance in each row.

### 4.6 Cost-benefit analysis

We conduct a qualitative analysis to further distinguish our proposed algorithm from existing feature selection algorithms. We compare our feature selection methodology with a non-exhaustive list of existing solutions, focusing on two primary criteria: interpretability and computational efficiency. To qualitatively evaluate the interpretability dimension, we distinguish between the set of mined features and the direction of influence. To characterise the set of mined features, we use the same naming convention of Kursa et al. (2010), who differentiates between “all-relevant” and “minimal-optimal” feature selection algorithms. While “all-relevant” describes a feature set containing all important features, “minimal optimal” refers to a setting where the optimal subset of features for a given classifier is mined. Table 6 provides an overview of the interpretability aspect. While all classifier-based feature selection solutions, except Boruta, represent minimal-optimal solutions, all filter methods are all-relevant. The direction of influence a feature has on the classification task depends on the chosen model. Our approach is the only solution offering a directed impact quantification with an all-relevant feature set. While this approach might yield a lot of statistically significant features, it is important for exploratory data analysis, where all features that contribute to a given classification problem are important and not just an inferred subset from a covariance matrix.

**Table 6:** Interpretability Analysis of feature selection methods for sequential patterns.

**Algorithm**	**Category**	**Interpretability**	
		**Feature set**	**Influence direction**
Mutual information	Filter	All-relevant	No
Chi-squared	Filter	All-relevant	No
LASSO^1^	Embedding	Minimal-optimal	Yes
Tree-based FI^2^	Embedding	Minimal-optimal	No
Forward selection	Wrapper	Minimal-optimal	Depends on model
Backward selection	Wrapper	Minimal-optimal	Depends on model
mRMR	Hybrid	Minimal-optimal	No
Boruta	Hybrid	All-relevant	No
Our approach	Hybrid	All-relevant	Yes

^1^Least absolute shrinkage and selection operator (L1 regularization).

^2^Feature importance (based on information gain).

Regarding computational efficiency, we differentiate between the maximum memory consumption and the time required to select the feature. Since our primary objective is to identify and interpret sequential patterns, we focus solely on mining them and their subsequent feature selection process. In particular, we compare the feature selection process as outlined in Algorithm 1 with the alternative approach of first mining the sequential patterns with the PrefixSpan algorithm and then selecting the important sequential patterns, for example, by means of the feature importance of a random forest or the mutual information criterion. This contrasts with Subsection 4.4, where we only compare the delta confidence measure with existing feature selection solutions and not the entire process.

**Figure 8:**
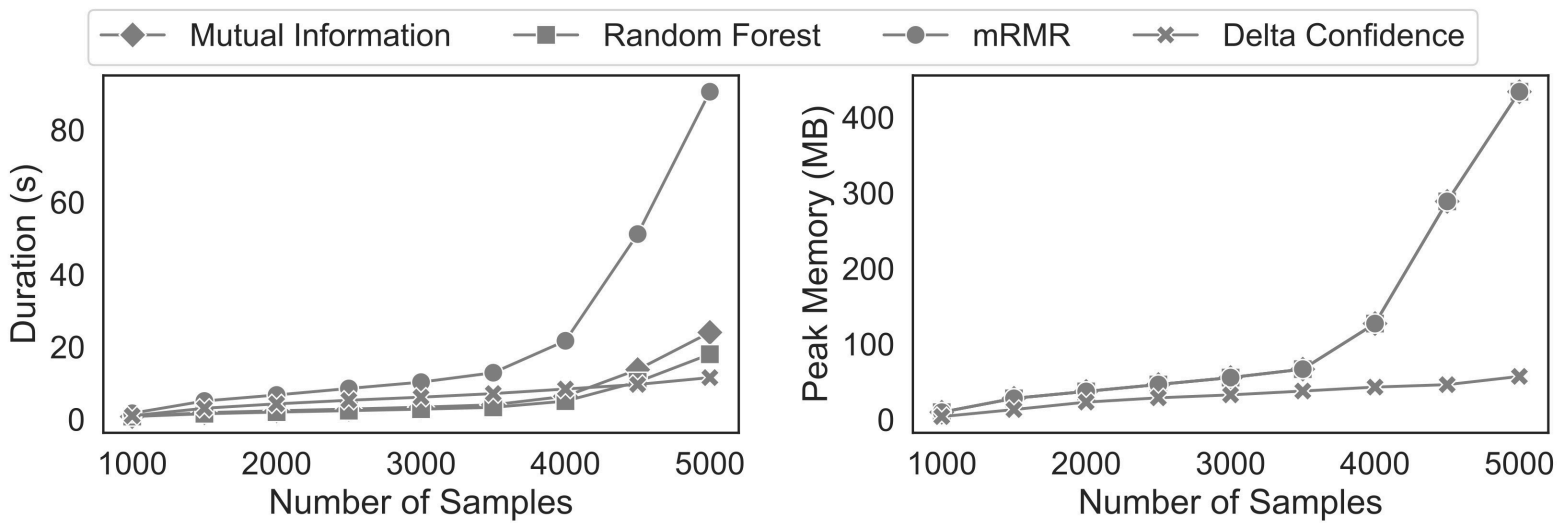
Computational efficiency analysis of feature selection methods for sequential patterns.

Figure 8 illustrates the time and peak memory required for increasing sample sizes of the synthetic dataset. As expected, mRMR is the most computationally expensive method in processing time since it incrementally looks for the sequential pattern that minimises the redundancy of the already existing features. While the random forest and mutual information show the fastest execution times for a low number of sequences, the delta confidence measure becomes relatively faster for an increasing number of sequences until it becomes the quickest feature selection method. We attribute this performance difference to two counteracting forces. First, for a sufficiently high bootstrap rate, mining sequential patterns multiple times on bootstrapped datasets generally takes longer than mining them on the full dataset. Second, with the delta confidence measure, it is not necessary to explicitly pass the mined sequences to another feature selection model. However, the random forest feature importance and the mutual information criterion require a separate projection of the mined sequential pattern on the observations. This step is computationally expensive since it involves checking for each sequential pattern if it is contained in an observation. Since this effect depends on the sample sizes and the number of sequential patterns, which increases with increasing sample sizes, the resulting computational time complexity is bilinear. Given that the PrefixSpan algorithm scales linearly with increasing sample sizes (Pei, 2001), our proposed delta confidence criterion scales better for large sample sizes. In terms of peak memory consumption, our delta confidence criterion avoids the costly creation of a one-hot encoded feature matrix, which is needed for subsequent machine learning models to estimate the importance of each sequential pattern. Given that this one-hot encoded matrix requires the majority of the memory, the peak consumption of the mRMR, random forest and the mutual information criterion overlap.

## 5 Discussion

In this work, we investigated whether we can correlate unsupervised SPM with supervised classification to enhance the selection of important sequences (RQ1) and how robust such a solution is compared to existing feature selection methods (RQ2). Our new feature selection algorithm successfully meets both criteria by using confidence scores from unsupervised SPM. It calculates differences for each class and assesses how much a sequence influences the classification task. Our correlation analysis shows a positive and statistically significant linear correlation with the target variable, indicating its usefulness for selecting subsequences. The subsequent analyses further confirm its robustness for feature selection and positions the delta confidence criterion as a viable alternative interesting measure. In particular, its high class specificity, its complementary information to statistical significance testing and its invariance to the imbalance in rule frequencies in comparison with the phi coefficient and 1-quality highlight its usefulness. Furthermore, in terms of feature selection, the delta confidence criterion obtains equal or better results on the synthetic and churn datasets than traditional feature selection criteria, such as mRMR, mutual information and random forest feature importance. When comparing interpretable machine learning solutions based on the memory-intensive one-hot encoding of the sequential patterns, our approach shows better peak memory consumption and better runtime statistics for an increasing number of sequences.

Our findings have multiple practical and theoretical implications. First, our robust feature selection algorithm offers a valuable alternative to existing machine learning-based methods that focus on identifying minimal-optimal subsets. In contrast, we emphasise an all-relevant feature set, similar to Boruta (Kursa et al., 2010), shifting the focus from purely predictive performance to a deeper understanding of the data, thus enriching exploratory data analysis. Additionally, among various interestingness measures in statistical sequential pattern mining, the delta confidence criterion stands out for its linear correlation with the target variable and high local class specificity. These properties make it particularly useful for practitioners seeking to identify meaningful patterns more effectively. Moreover, due to its direct interpretability, it eliminates the need for *post hoc* explanation tools, such as SHAP (Lundberg and Lee, 2017) or LIME (Ribeiro et al., 2016). These interpretability layers are typically fitted on potentially erroneous predictions from black-box models, which may lead to flawed estimations of importance. Accurate interpretability is particularly important in high-stakes domains, such as finance (Rudin, 2019) and medicine (Žlahtič et al., 2023), where our method may increase trust and transparency (Adewale Abayomi Adeniran et al., 2024; Rane et al., 2023). On a more general note, our work on mining sequential patterns can also be applied to other areas, such as rare pattern mining, which has only been done on itemsets. Furthermore, it is easy to interpret, making it an easy metric to report to stakeholders, and does not require heavy upskilling as opposed to deep learning technologies, which introduce dependencies on third-party providers and monetary dependencies in the data management lifecycle (Borah et al., 2022). Lastly, we challenge the notion that multiple SPM rounds always lead to longer runtimes in the overall feature selection process. This finding encourages researchers and practitioners to rethink the end-to-end process when mining the most important sequential patterns.

Despite the overall positive results, the findings also highlight some limitations of our approach and the selection of sequential patterns for classification problems in general. Table 5 indicates that, in some scenarios, especially when the sparsity of the obtained sequences is high, it might be sufficient to focus only on the single events and not the sequences themselves when it comes to classification performance. However, by relying on single events rather than sequences, information about important temporal relations is lost. Furthermore, the delta confidence values in Figure 1 display a varying degree of variance depending on the dataset, which can be attributed to aleatoric and epistemic uncertainty (Hüllermeier and Waegeman, 2021; Gal, 2016). In addition to aleatoric uncertainty, which is induced by randomness in data and is therefore irreducible, our approach introduces epistemic uncertainty by bootstrapping and the delta confidence measure estimation itself, which can have multiple values for the same sequence. However, compared to traditional approaches, this additional (epistemic) uncertainty does not necessarily result in a worse feature selection performance as Figure 7 shows. Further work is needed to validate this finding empirically. Another potential shortcoming concerns the number of mined sequences. While advantageous in exploratory data analysis, the all-relevant feature selection property of our approach leads to a relatively large subset of important and potentially correlated sequences. One could leverage local and global correlations as proposed by Chen et al. (2024) to decorrelate the sequences while maintaining a representative subset. Whereas global correlations, which are based on the lift measure for SPM, ensure that the overall sequence is relevant for the whole dataset, the local correlation ensures that the connection between the antecedent and the consequent is strong. By combining and setting adequate thresholds, practitioners can reduce the number of sequential patterns beyond their statistical significance for the classification problem.

Another limitation of this work is its narrow focus on interpretable machine learning for SPM, with the primary goal of identifying important sequences. Given this objective, we use one-hot encoding since it is the standard approach in interpretable machine learning, ensuring direct traceability between input features and model decisions. While alternative encoding methods, such as tf-idf, Markov Chains (Gagniuc, 2017), Network Motifs (Masoudi-Nejad et al., 2012) and learned sequence representations via recurrent neural networks, could be integrated into xAI frameworks, they introduce limitations that make them less suitable for our goal. For instance, tf-idf ignores the temporal structure of the input sequence. Similarly, Markov Chains create a memoryless transition matrix, abstracting away specific sequence occurrences. Network Motifs focus on higher-order structural patterns in networks, which may overlook fine-grained sequential dependencies, while learned sequence representations, such as embeddings, introduce black-box transformations that hinder direct interpretability. Furthermore, we only compare the delta confidence measure with other existing interesting measures, but do not compare the overall framework as illustrated in Algorithm 1 with other SPM methods that leverage statistical testing.

Further research should focus on three significant areas. First, future work must compare our proposed feature selection algorithm with recent advancements in deep learning, especially in graph neural networks and explainable artificial intelligence, and algorithms from subgroup discovery for larger datasets. This would provide additional guidance on when to choose which method, based on runtime-accuracy-interpretability trade-offs. In particular, future work needs to investigate how the interpretability of the delta confidence criterion compares against existing explainability approaches, to assess its advantages and limitations from a user-understandability perspective. Given the relatively small size of the datasets employed in this study, the application of deep learning methods could be considered too complex a solution for the purpose; however, for larger datasets, a comparative evaluation of the proposed feature selection algorithm with techniques, such as GNNexplainer (Ying et al., 2019) or WindowSHAP (Nayebi et al., 2023), would be warranted. While the GNNexplainer extracts subgraphs with a high contribution to the target variable, WindowSHAP can explain predictions of time series models. Yet, these attribution scores of WindowSHAP need additional analysis to identify their corresponding global feature importance. Also, further comparison with subgroup discovery algorithms, such as LAMP (Terada et al., 2013b), WYLight (Llinares-López et al., 2015) and SPASS Dalleiger and Vreeken (2022), would greatly enhance comparability in terms of runtime and selected features. Second, besides comparing to other existing algorithms, further research is required to investigate and enhance the robustness of our approach. This involves, in particular, experiments with severe class imbalance, rare but important patterns and research on decreasing the epistemic uncertainty of our delta confidence criterion. These findings would significantly improve the practitioners' understanding and usability of our approach in noisier datasets. Thirdly, to guarantee the extensive adoption and scalability of our proposed algorithm, it is imperative to enhance its computational efficiency. Although the current implementation has been adequate to demonstrate the efficacy of the delta confidence criterion, the substitution of the maximum sequence and minimum support thresholds in favour of an enhanced Branch-and-Bound algorithm analogous to LAMP can augment its efficiency and generalisability (Terada et al., 2013a; Minato et al., 2014).

## 6 Conclusion

This work presents a novel feature selection technique that selects informative sequences from discrete sequential data. Despite their prominence in practical applications, selecting informative subsequences for classification tasks is underexplored in academia. Existing machine learning literature mainly covers feature selection and extraction for numerical time series and tabular data. In contrast to computationally intensive deep learning approaches, our work offers a simple and easy-to-understand approach to selecting informative subsequences for classification problems. Our evaluation of three different datasets shows that our feature selection criterion of the selected features correlates strongly with the associated classification target, implying that our feature selection criterion helps select features and can also be used to reliably estimate the impact a sequence has on a classification problem. The comparison with existing interesting measures for subgroup discovery also shows a high correlation with our delta confidence criterion, which offers complementary insights to existing subgroup discovery measures and helps practitioners uncover hidden sequential patterns in their data. Compared with one-hot encoded feature selection of sequential patterns, our approach is more memory efficient and scales better for an increasing number of sequences. Future research might leverage the recent developments of interpretable time series classification with deep learning and focus on extracting interpretable sequences from their predictive explanations.

## Data Availability

The non-synthetic datasets analyzed in this study are available in online repositories. The code used for analysis is available on GitHub at https://github.com/alexandergrote/cts. The non-synthetic data are available via at https://1drv.ms/f/c/e6dfa373b2b71977/Er8YtL3HulpKikuQCZGxKbUBlKdSuSUlnHBsY78Ne0-Hmg?e=Bp9Mxh Both repositories provide the materials necessary to reproduce the findings reported in this study.

## Footnotes

^1^We use the terms “informative”, “interesting” and “important” interchangeably to describe the sequences that carry information about the underlying classification problem.

^2^This task is studied under various names in the literature, including subgroup discovery (Atzmueller, 2015), discriminative pattern mining (Cheng et al., 2008), and contrastive pattern mining (Bay and Pazzani, 2001). Accordingly, we use these terms interchangeably in this work.

^3^We have verified the overall trends for the synthetic and churn datasets as well. While the synthetic dataset exhibits trends identical to those observed in the malware dataset, the churn dataset does not produce a symmetric distribution of delta confidence values as shown in Figure 1, limiting the interpretability of the full range of relationships. To conserve space, we present visualisations of the subgroup measures exclusively for the malware dataset.
